# Extracellular vesicles derived from umbilical cord mesenchymal stromal cells alleviate pulmonary fibrosis by means of transforming growth factor-β signaling inhibition

**DOI:** 10.1186/s13287-021-02296-8

**Published:** 2021-04-12

**Authors:** Liyan Shi, Jing Ren, Jiping Li, Dongxu Wang, Yusu Wang, Tao Qin, Xiuying Li, Guokun Zhang, Chunyi Li, Yimin Wang

**Affiliations:** 1 China-Japan Union Hospital of Jilin University, 126 Xiantai St., Changchun, 130033 Jilin China; 2 Institute of Antler Science and Product Technology, Changchun Sci-Tech University, Changchun, 130600 Jilin China; 3 School of Ecology and Environment, Northwestern Polytechnical University, 1 Dongxiang Rd, Xi’an, 710129 Shaanxi China; 4 Institute of Special Animal and Plant Sciences, Chinese Academy of Agricultural Sciences (CAAS), 4899 Juye St., Changchun, 130112 Jilin China

**Keywords:** Extracellular vesicles, Umbilical cord-derived mesenchymal stromal cells, Pulmonary fibrosis, Transforming growth factor-β

## Abstract

**Background:**

Pulmonary fibrosis (PF), the end point of interstitial lung diseases, is characterized by myofibroblast over differentiation and excessive extracellular matrix accumulation, leading to progressive organ dysfunction and usually a terminal outcome. Studies have shown that umbilical cord-derived mesenchymal stromal cells (uMSCs) could alleviate PF; however, the underlying mechanism remains to be elucidated.

**Methods:**

The therapeutic effects of uMSC-derived extracellular vesicles (uMSC-EVs) on PF were evaluated using bleomycin (BLM)-induced mouse models. Then, the role and mechanism of uMSC-EVs in inhibiting myofibroblast differentiation were investigated in vivo and in vitro.

**Results:**

Treatment with uMSC-EVs alleviated the PF and enhanced the proliferation of alveolar epithelial cells in BLM-induced mice, thus improved the life quality, including the survival rate, body weight, fibrosis degree, and myofibroblast over differentiation of lung tissue. Moreover, these effects of uMSC-EVs on PF are likely achieved by inhibiting the transforming growth factor-β (TGF-β) signaling pathway, evidenced by decreased expression levels of TGF-β2 and TGF-βR2. Using mimics of uMSC-EV-specific miRNAs, we found that miR-21 and miR-23, which are highly enriched in uMSC-EVs, played a critical role in inhibiting TGF-β2 and TGF-βR2, respectively.

**Conclusion:**

The effects of uMSCs on PF alleviation are likely achieved via EVs, which reveals a new role of uMSC-EV-derived miRNAs, opening a novel strategy for PF treatment in the clinical setting.

**Supplementary Information:**

The online version contains supplementary material available at 10.1186/s13287-021-02296-8.

## Background

Pulmonary fibrosis (PF), the end point interstitial lung diseases, is described as the deposition of excessive collagen and other extracellular matrix molecules within the alveolar septa (thickening of the septal interstitium), with or without structural lung damage and fibrotic masses, depending on the severity [1–3]. Currently available treatments for PF fail to significantly increase the survival rate of patients, especially in the case of the current pandemic of COVID-19. One currently assumed pathogenesis of PF is fibroblast-to-myofibroblast transition (FMT) initiated and driven by the transforming growth factor-β (TGF-β) signaling pathway [4, 5]. When the alveolar epithelium is injured, activated fibroblast proliferation and macrophage infiltration produce a higher amount of TGF-β [4, 5]. Then, the downstream genes of the TGF-β signaling pathway, including TGF-β receptor and Smad, are activated, which leads to the extensive expression of α-smooth muscle actin (α-SMA) and collagen, thereby promoting FMT [6]. Therefore, targeting the TGF-β signaling pathway to inhibit FMT is considered a practical therapeutic strategy for PF.

In recent years, cell therapy based on mesenchymal stromal cells (MSCs) has been widely used to treat various diseases [7] and reported excellent therapeutic effects on tissue injuries, including the bone [8], cartilage [9], skin [10–12], brain [13], liver [14], lung [15–17], and heart [18–20]. Among these diseases, MSCs may elicit a superior therapeutic response on lung injury because administered MSCs first reach the lung after being transplanted through a vein [15, 21]; especially under the current situation, researchers have proposed using MSCs to treat PF caused by COVID-19 [22]. In addition, MSCs exhibit anti-fibrosis activities in scar healing of wound [10–12] and organ fibrosis [14–17], which would be relevant to PF. MSCs are usually obtained from the adult bone marrow, umbilical cord blood, adipose tissue, and placenta [23]. However, the differentiation potential of MSCs decreases as the donor’s age increases, limiting their applications [24–26]. Umbilical cord-derived MSCs (uMSCs) have high cell differentiation abilities and thus are preferred for transplantation in this regard [10, 26, 27].

uMSCs exert a therapeutic effect on PF due to their ability to homing to the injured lung and differentiate into specific cell types needed for the repair [28]. However, based on the reports [10, 26, 28–30], only few transplanted MSCs could survive in the injury site; thus, the therapeutic effects of MSCs are generally believed to be achieved by their immunomodulatory effects (interacting with cells of the adaptive and innate immune system) and trophic benefit (neovascularization, recruitment of cells beneficial for tissue repair or activation of tissue intrinsic progenitor cells). Most of these effects may be mediated by their secretome that includes secreted soluble factors and extracellular vesicles (EVs). We consider that uMSCs can transfer functional RNAs and proteins to other cells through EVs, with beneficial effects on tissue repair after lung injury. EVs are essential for cell functions and are considered as a novel paracrine factor released by cell outward budding [31, 32]. Although the therapeutic effect of uMSC-EVs has been observed in a previous study [33], the underlying mechanism remains to be elucidated.

The present study investigated the antifibrotic effect of uMSC-EVs using a bleomycin (BLM)-induced mouse model, and our results demonstrated that uMSC-EVs alleviated PF through inhibiting the TGF-β signaling pathway. MiR-21 and miR-23 are carried by uMSC-EVs as crucial elements contributing to anti-myofibroblast differentiation by downregulating TGF-β2 and TGF-βR2 expression. Overall, we believe that our results have opened up a new avenue for using uMSC-EVs to treat PF in clinics.

## Materials and methods

### Cell culture

Human uMSCs were obtained from the China-Japan Union Hospital, and characterization using surface antigen (Table S1) was performed via flow cytometry (FCM) and immunofluorescence (IF) staining. WML2 fibroblast cells were obtained from the Basic Medical Science of Jilin University. All cells used in this study were in passage 3–5 and cultured in Dulbecco’s modified Eagle’s medium (DMEM; BI, Israel) + 10% fetal bovine serum (FBS; BI, Israel) in a standard incubator with 37 °C, 5% CO_2_, and 80% humidity.

### EV isolation

uMSCs were grown to 80% density in DMEM with FBS and then replaced with serum-free medium (BI) and cultured for 48 h. The culture supernatant was collected, filtered through a 0.1-μm filter device, and then ultracentrifuged at 100,000*g* for 3 h. The precipitates (EVs) were washed thrice using PBS. uMSC-EVs were confirmed using transmission electron microscopy and the exosomal markers CD9, CD63, and TSG101 (Beyotime, China) through western blot. The EVs were stored at − 80 °C until use.

### Treatment of the cultured cells

WML2 fibroblast cells (1 × 10^5^ cells/mL) were seeded in a 24-well plate and then cultured in DMEM + 10% FBS (+TGF-β1; 5 ng/mL) for 48 h to induce FMT in vitro. uMSC-EVs (10 ng/mL) were synchronously used to observe the inhibition effect on the treated cells. MiR-21 mimics (50 nM) or miR-23a mimics (50 nM) were also used to observe the inhibition effect on the treated cells, and Lipofectamine 3000 (Thermo, USA) was used for the transfection of mimics; a nonsense sequence was used as the negative control. The expression levels of α-SMA, TGF-β2, and TGF-βR2 in the cells were determined using quantitative real-time polymerase chain reaction (qRT-PCR) and IF staining analyses.

### IF staining

Cells were fixed in 4% paraformaldehyde (30 min), permeabilized with 0.1% Triton X-100 (15 min), blocked using 3% BSA (30 min), incubated with primary antibodies at 4 °C (12 h), and then stained with AF 647- or AF 488-labeled secondary antibodies (Thermo, USA). The primary antibodies used in the study were anti-Ki67 (bs-23105R, Bioss, China), anti-α-SMA (bsm-33188M, Bioss, China), anti-fibronectin (anti-Fn; bs-0666R, Bioss, China), anti-transforming growth factor-β1 (anti-TGF-β1; bsm33345M, Bioss, China), anti-transforming growth factor-β2 (anti-TGF-β2; bs-20412R, Bioss, China), anti-transforming growth factor-β3 (anti-TGF-β3; AF8142, Beyotime, China), anti-transforming growth factor-β receptor type I (anti-TGF-βR1; bs0638R, Bioss, China), and anti-transforming growth factor-β receptor type II (anti-TGF-βR2; AF8151, Beyotime, China). Nuclear staining was performed with DAPI (Beyotime, China). Images were examined under a fluorescence microscope (BX63, Olympus, Japan).

### Mouse model

All procedures that were carried on mice were approved by the Administration Committee of the Institute of Antler Science and Product Technology of Changchun Sci-Tech University (Approval No.: IASPT202006). Adult male C57BL/6 mice (6–8 weeks old) were purchased from Liaoning Changsheng Biotechnology Co., Ltd. (Benxi, China). Mice were intratracheally instilled with a single dose of BLM (3 U/kg) on day 0 to induce PF. The control group was treated similarly as above but BLM was replaced with PBS. BLM-induced mice were randomly allocated into three groups: (1) uMSC (1 × 10^6^), (2) uMSC-EVs (20 μg) in 100 μL of PBS, and (3) equal volumes of PBS. The treatments were carried out via intravenous injection on days 7 and 21, respectively. All mice were euthanized on day 35 and lung was collected. Each lung was divided into two parts: one part was fixed in 10% formaldehyde solution for 48 h, and the other was stored at − 80 °C.

### uMSC- and uMSC-EV-tracing in PF mice

First, uMSCs and uMSC-EVs were labeled with PKH67 for living cell staining following the manufacturer’s instructions (Umibio Co. Ltd., China). Then, the PKH67-labeled uMSCs or uMSC-EVs were injected via mouse tail veins on day 7 after BLM stimulation. The lung tissues were sampled on days 1, 4, and 7 and after treatment with uMSCs or uMSC-EVs. The lung tissues were embedded in OCT (Tissue-Tek O.C.T.), cut at 5 μm, stained with DAPI (Beyotime, China), and then examined/photographed under a fluorescent microscope (BX63, Olympus, Japan). Hematoxylin and eosin (H&E) staining were also performed.

### Hydroxyproline assay

The content of collagen in the lung homogenate was analyzed using a hydroxyproline (HYP) assay kit (Solarbio, China).

### Histology and immunostaining

The right lung tissues were embedded in paraffin and sliced into 4-μm sections. For histologic evaluation, the sections were stained with HE to observe the structure and with Masson to detect collagen deposits. Three pathologists scored the development of lung lesions. The sections were stained via IF, referred to as cell immunostaining, for analysis of garget gene expression. Sections were photographed using Leica Microsystems (Leica DMi8, Germany) and fluorescent microscope (BX63, Olympus, Japan).

### qRT-PCR

The total RNA of the cells and lung tissues was isolated using Trizol reagent (Sigma-Aldrich, USA) and then reverse transcribed into cDNA using a cDNA synthesis kit (TaKaRa, Japan). SYBR premix (Roche, Switzerland), primers, and cDNA were combined on qRT-PCR Detection System (qTOWER ^3^G, Germany). qRT-PCR of miRNA was performed using the miScript SYBR Green PCR Kit (Qiagen, China) in accordance with the manufacturer’s instructions. All results were normalized to U6 small RNA levels measured with the Hs_RNU6B_2 miScript Primer Assay kit (Qiagen). The primers are listed in Table S2.

### Statistical analysis

Data are expressed as mean ± SEM. Comparison of variables between multiple groups was performed using one-way ANOVA with Tukey post hoc test: *p < 0.05, **p < 0.01, and ***p < 0.001.

## Results

### uMSC-EV treatment alleviated pulmonary fibrosis in the BLM-induced mice

**Fig. 1:**
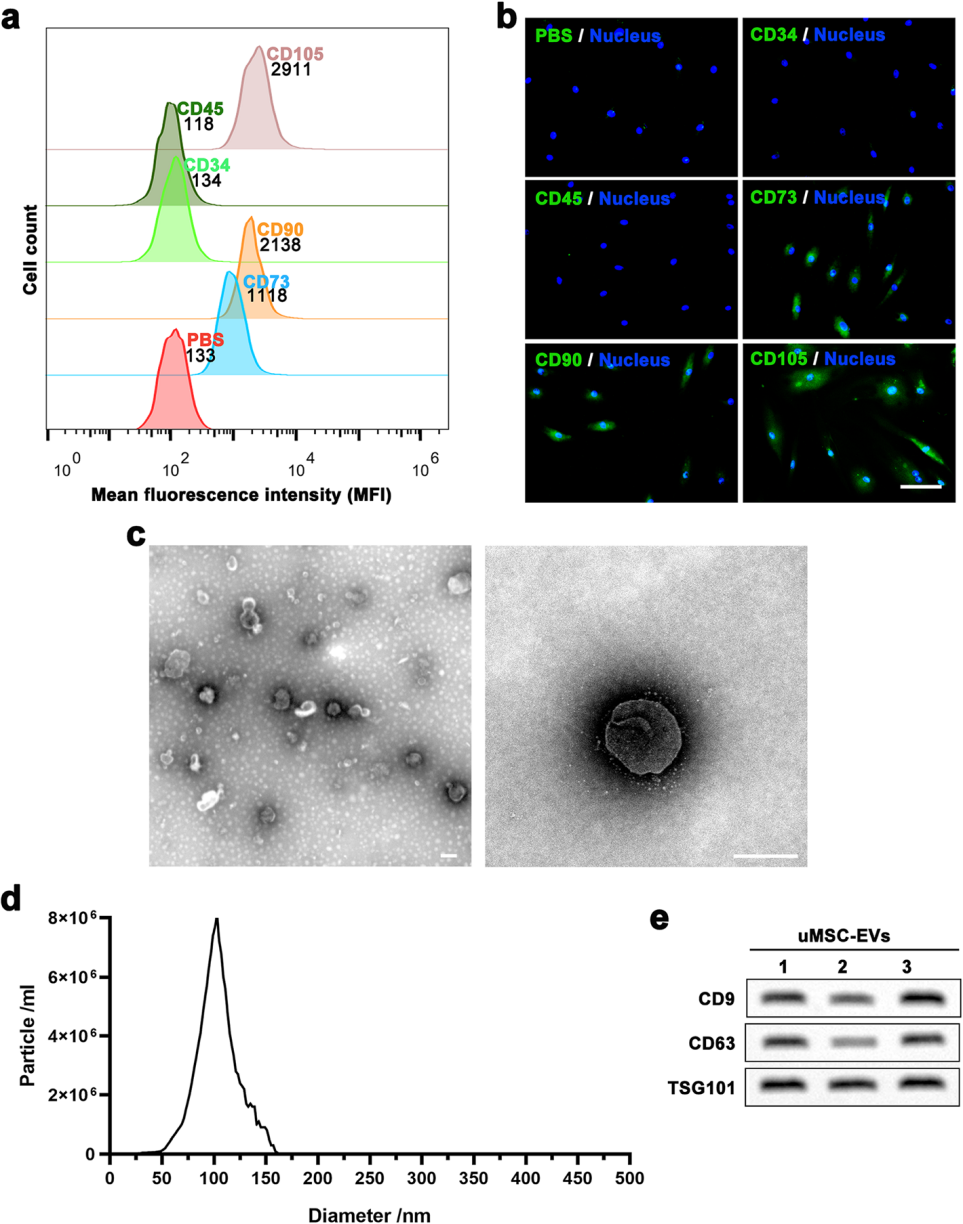
Identification of uMSCs and characterization of uMSC-EVs. **a**, **b** The surface antigen (CD34, CD45, CD73, CD90, and CD105) in uMSCs was detected using FCM and IF staining, scale bar = 100 μm. **c** Morphological characteristics of uMSC-EVs were observed via transmission electron microscopy, scale bar = 100 nm. **d** Exosomal markers (CD9, CD63, and TSG101) of uMSC-EVs were detected using western blot assay. **e** uMSC-EV particle size was detected using NanoSight. FCM, flow cytometry; IF, immunofluorescence staining; uMSCs, umbilical cord-derived mesenchymal stromal cells; uMSC-EVs, umbilical cord-derived mesenchymal stromal cell-derived extracellular vesicles

uMSCs were confirmed using FCM and IF staining, and the results showed that uMSCs were positive to CD73, CD90, and CD105, negative to CD34 and CD45 (Fig. 1a, b). uMSC-EVs were confirmed using a transmission electron microscope, NanoSight, and western blot analysis. Results showed that the particle size of uMSC-EVs was between 50 and 160 nm (Fig. 1c, d); uMSC-EVs were intensively stained with exosomal markers CD9 and CD63 and TSG101 (Fig. 1e).

**Fig. 2:**
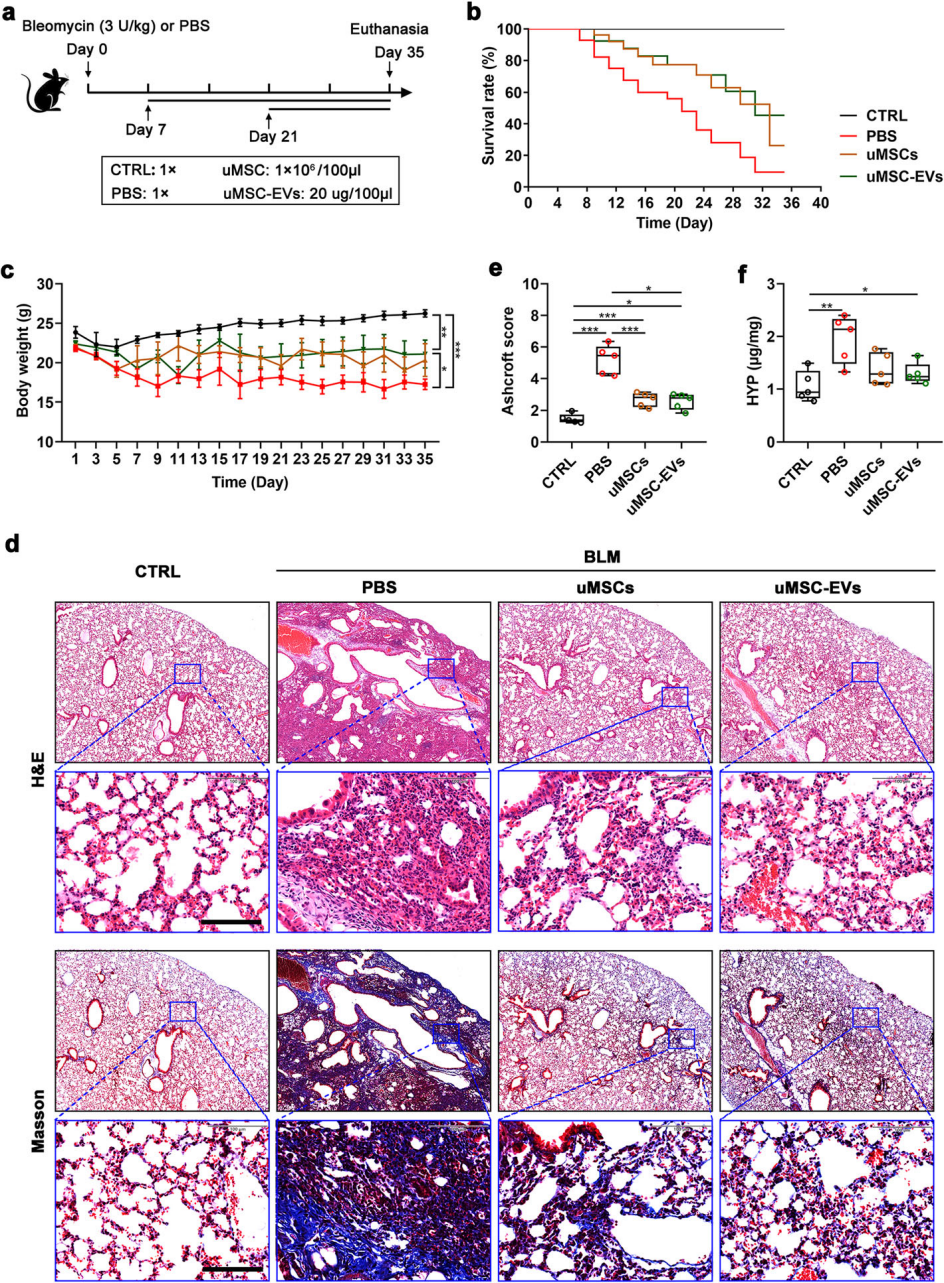
uMSC-EVs alleviated BLM-induced pulmonary fibrosis. Mice were intratracheally instilled with a single dose of BLM (3 U/kg), and then each mouse was randomly allocated to receive either uMSC (1 × 10^6^) or uMSC-EVs (20 μg) in 100 μL of PBS or equal volumes of PBS alone via intravenous injection on days 7 and 21; normal mice were served as control. **a** Schematic of the experimental design. **b**, **c** Survival rate and change of body weight after BLM treatment. **d** H&E and Masson staining was subjected for lung tissue sections. Scale bar = 100 μm. **e** Ashcroft score of histological images was determined by pathologists blind to the study design. **f** HYP content of lung tissue. Mean ± SEM; *p < 0.05, **p < 0.01, ***p < 0.001; *n* = 5. BLM, bleomycin; control (CTRL); H&E, hematoxylin and eosin; HYP, hydroxyproline

**Fig. 3:**
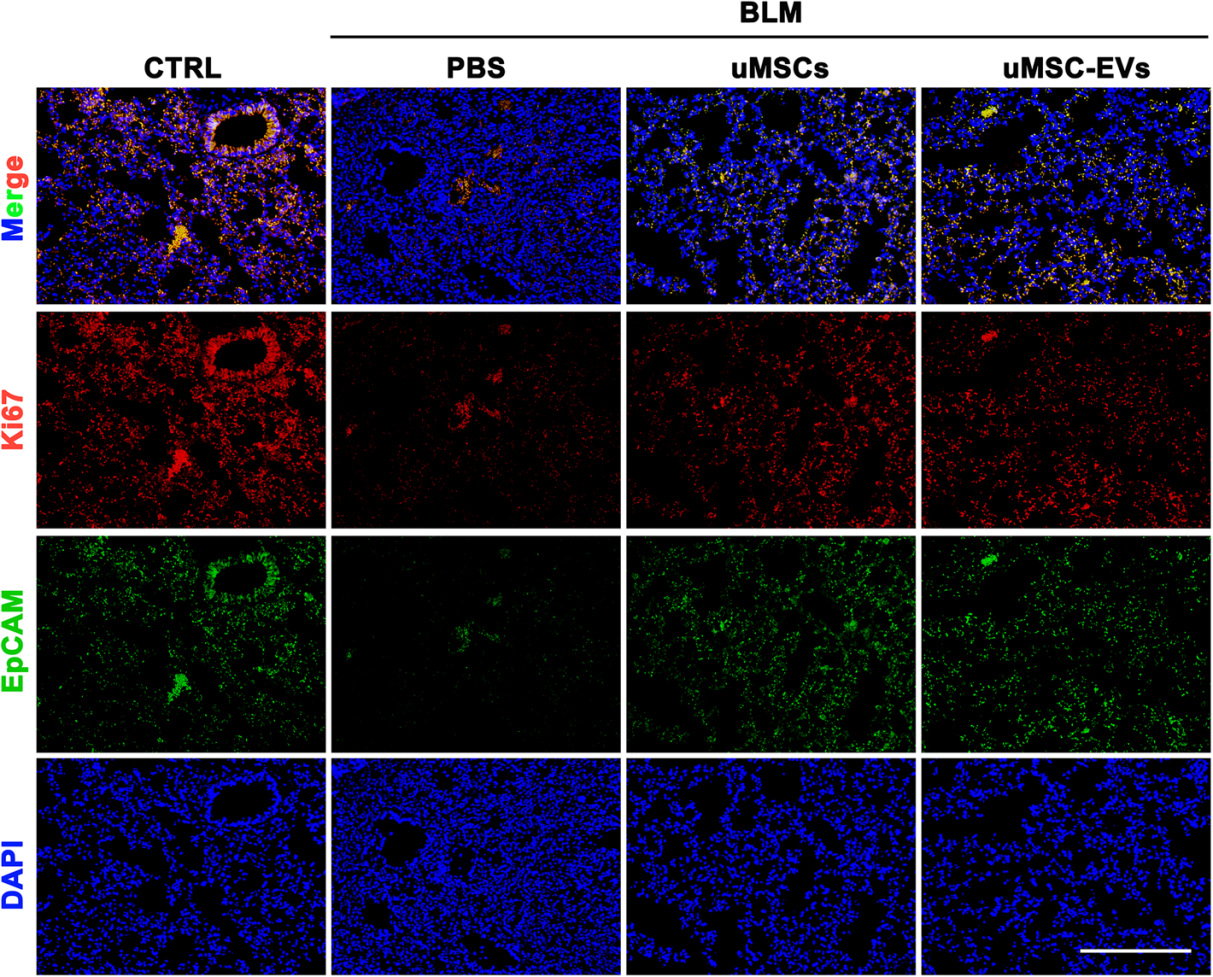
uMSC-EVs enhanced the proliferation of alveolar epithelial cells in BLM-induced pulmonary fibrosis. Expression levels of Ki67 (proliferating cell) and EpCAM (epithelial cell) were detected using IF staining. Scale bar = 200 μm

Mice were treated with uMSC-EVs through intravenous injection on days 7 and 21 after BLM administration to evaluate the effect of uMSC-EVs on PF (Fig. 2a). Results showed that BLM-treated mice had lower survival rate and body weight, damaged tissue architecture, and dense deposition of collagen. However, both uMSC and uMSC-EV treatments improved the survival rate and body weight of PF mice compared with the PBS treatment (Fig. 2b, c). Both the degree of lung tissue damage and collagen deposition were also improved by uMSC and uMSC-EV treatments compared with the PBS treatment (*p* < 0.05; Fig. 2d–f). The expression levels of Ki67 in lung tissues were investigated, and results showed that BLM treatment resulted in decreased Ki67 expression. Interestingly, Ki67 expression was strongly stimulated by the uMSC and uMSC-EV treatments compared with the PBS treatment; moreover, the Ki67^+^ cells and EpCAM^+^ (maker of epithelial cells) [34] cells were found to be overlapped in the lung tissue sections (Fig. 3). Notably, both uMSCs and uMSC-EVs showed no significant difference in therapeutic effects of the BLM-treated mice, suggesting that uMSCs can alleviate PF, at least in part via uMSC-EVs.

### uMSC-EVs suppressed myofibroblast differentiation via inhibiting the TGF-β signaling pathway in lung tissue of the PF mice

**Fig. 4:**
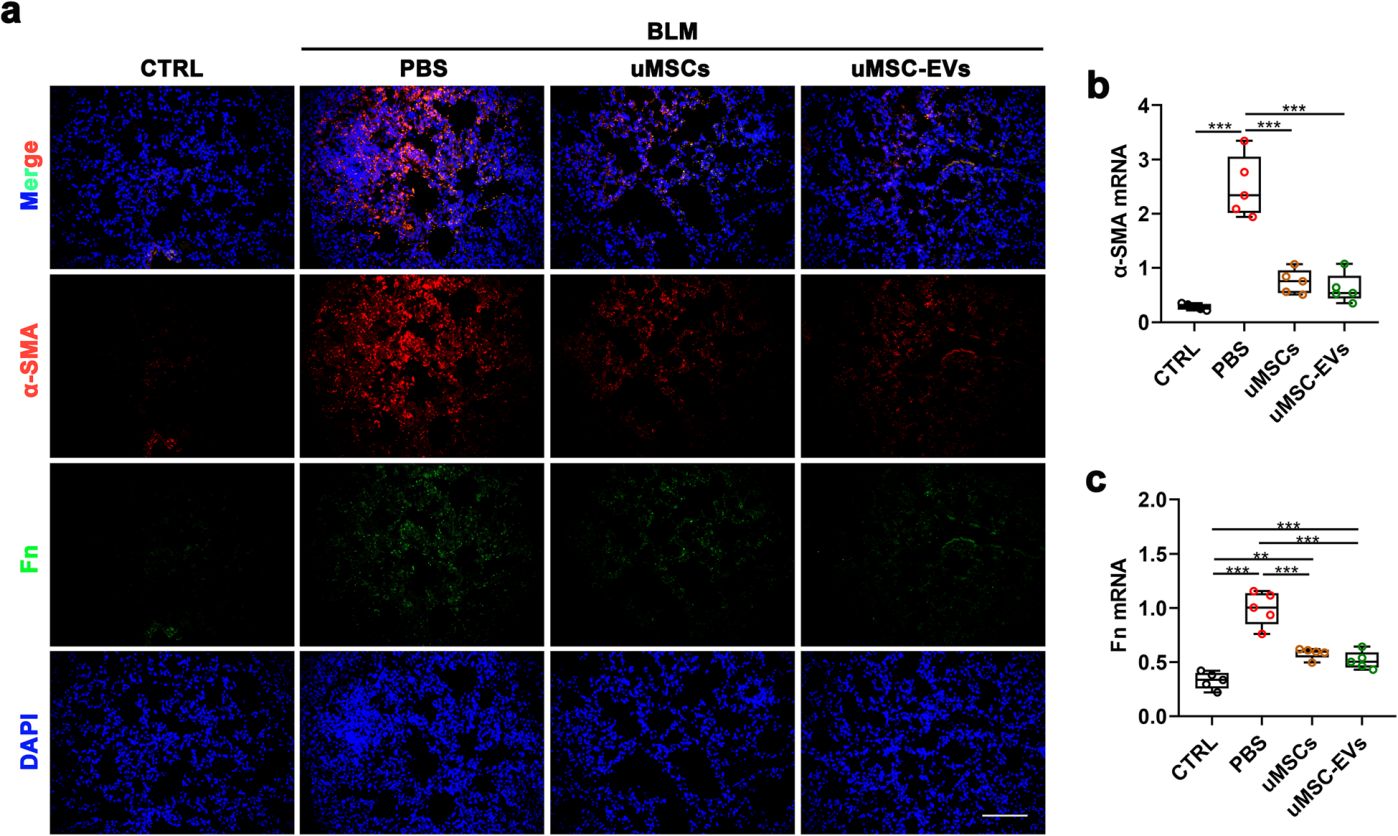
uMSC-EVs inhibited myofibroblast differentiation in lung tissues of BLM-induced mice. **a**, **b** Expression levels of α-SMA and Fn were detected using IF staining and qRT-PCR. Scale bar = 200 μm. Mean ± SEM; **p < 0.01, ***p < 0.001; *n* = 5. α-SMA, α-smooth muscle actin; Fn, fibronectin

**Fig. 5:**
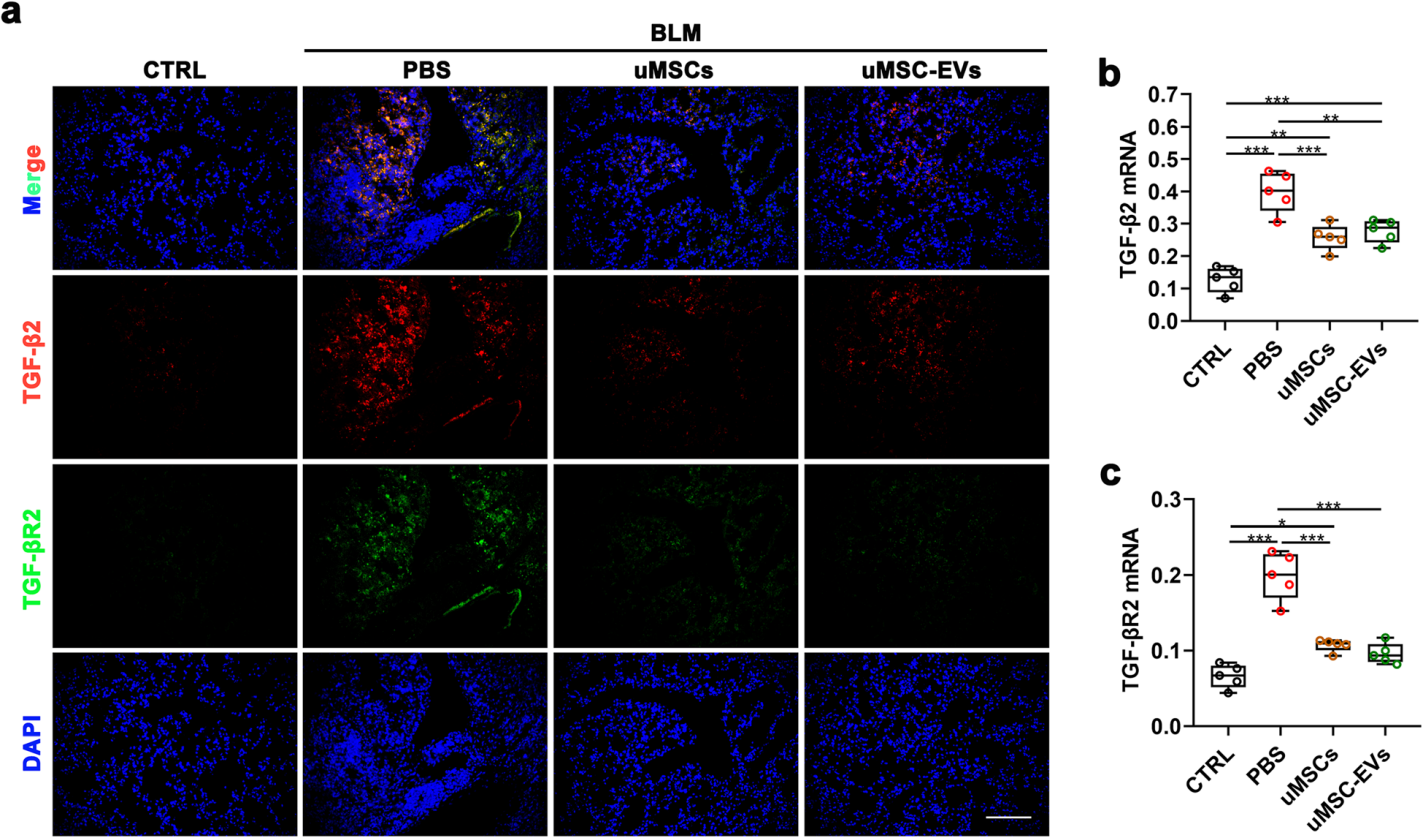
uMSC-EVs inhibited TGF-β2 and TGF-βR2 expression in lung tissues of BLM-induced mice. **a**, **b** Expression levels of TGF-β2 and TGF-βR2 were detected using IF staining and qRT-PCR. Scale bar = 200 μm. Mean ± SEM; *p < 0.05, **p < 0.01, ***p < 0.001; *n* = 5. TGF-β2, transforming growth factor-β2; TGF-βR2, transforming growth factor-β receptor type II

α-SMA and Fn are critical fibrotic markers of myofibroblasts and fibrotic diseases [5, 6]. The expression levels of α-SMA and Fn in the lung tissues were measured using IF staining and qRT-PCR analysis. As expected, α-SMA and Fn were highly expressed in the lung tissues of PF mice. Interestingly, these high expression levels of α-SMA and Fn were reduced with the uMSC and uMSC-EV treatments (Fig. 4). Furthermore, TGF-β2 and TGF-βR2, the upstream genes of α-SMA and Fn in the TGF-β signaling pathway [5, 6], were highly expressed in the lung tissues of PF mice. Consistently, both uMSC and uMSC-EV treatments also reduced high expression levels of these two genes (Fig. 5). Notably, there was no significant difference being detected between the uMSC and uMSC-EV treatments in the above gene expression in the lung tissue of BLM-treated mice. These results suggest that uMSC-EVs can inhibit myofibroblast differentiation likely through inhibiting the TGF-β signaling pathway in the lung tissue of PF mice.

### uMSC-EVs suppressed TGF-β1-induced myofibroblast differentiation in vitro

**Fig. 6:**
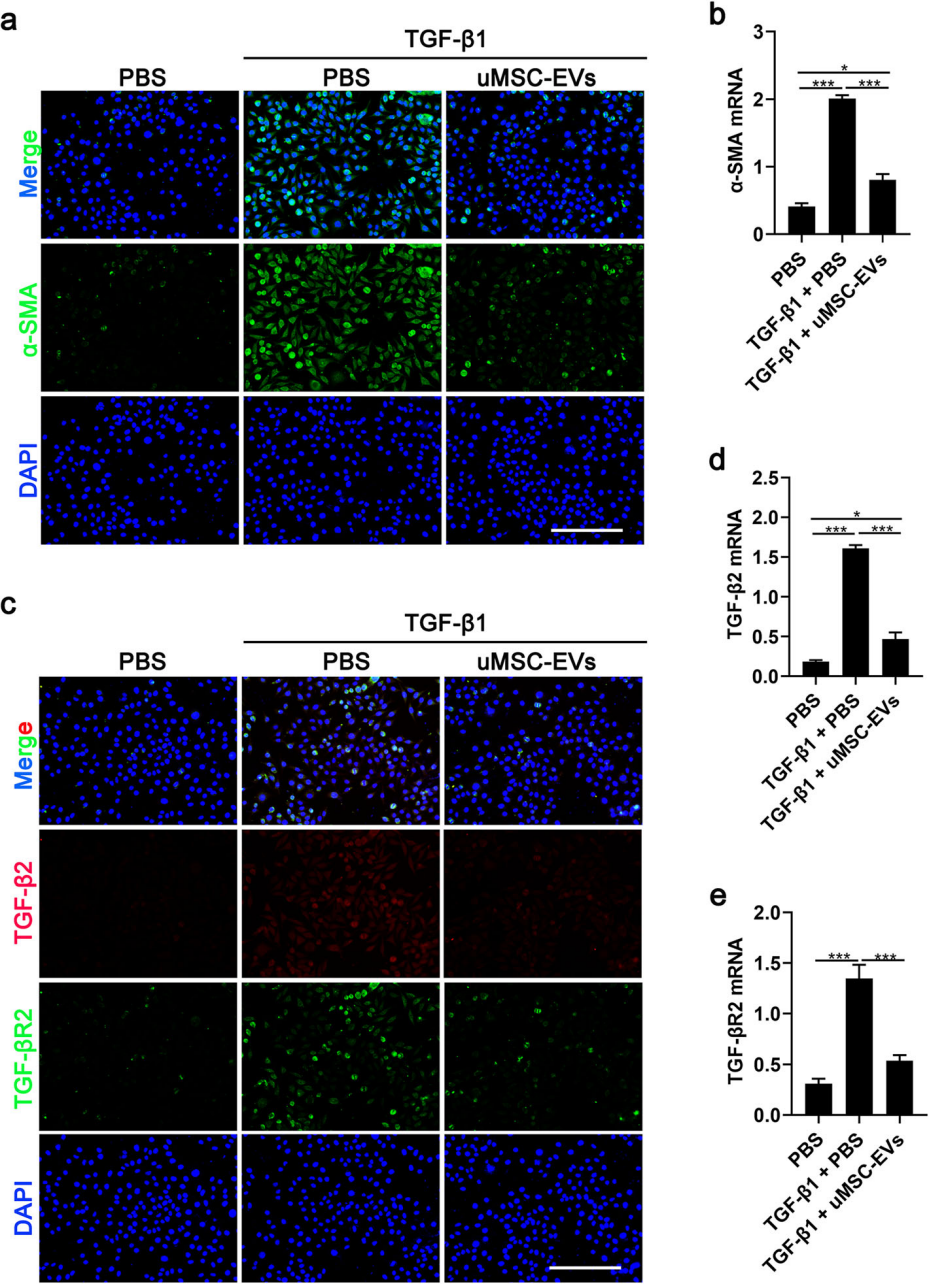
uMSC-EVs inhibited TGF-β1-induced myofibroblast differentiation in vitro. WML2 fibroblast cells were cultured for 48 h with TGF-β1 to induce myofibroblast differentiation, some of the cells were also treated with uMSC-EVs as indicated, then expression levels of α-SMA (**a**, **b**), TGF-β2 (**c**, **d**), and TGF-βR2 (**c**, **e**) were detected in different treatments using qRT-PCR and IF. Scale bar = 200 μm. Mean ± SEM; *p < 0.05, **p < 0.01, ***p < 0.001; *n* = 3. TGF-β1, transforming growth factor-β1

Lung fibroblast cells (WML2) were cultured in the presence of TGF-β1 to induce myofibroblast differentiation in vitro*,* through which the effect of uMSC-EV treatment on myofibroblast differentiation was verified. Cultured cells increased the expression level of α-SMA with increased dosage of TGF-β1 through IF staining and qRT-PCR, suggesting that the cell model is reliable (Fig. S1). Then, 10 ng/mL uMSC-EVs were added to TGF-β1 (5 ng/mL)-treated cells. The results of IF staining and qRT-PCR analysis showed that uMSC-EV treatment strongly inhibited the TGF-β1-induced high expression of α-SMA (Fig. 6a, b). The expression levels of TGF-β2 and TGF-βR2 in TGF-β1-induced WML2 fibroblast cells were also reduced with uMSC-EV treatment (Fig. 6c–e). The results indicate that uMSC-EVs could effectively suppress TGF-β-induced myofibroblast differentiation in vitro.

### uMSC-EV-specific miRNAs target TGF-β2 and TGF-βR2 to inhibit the TGF-β signaling pathway

**Fig. 7:**
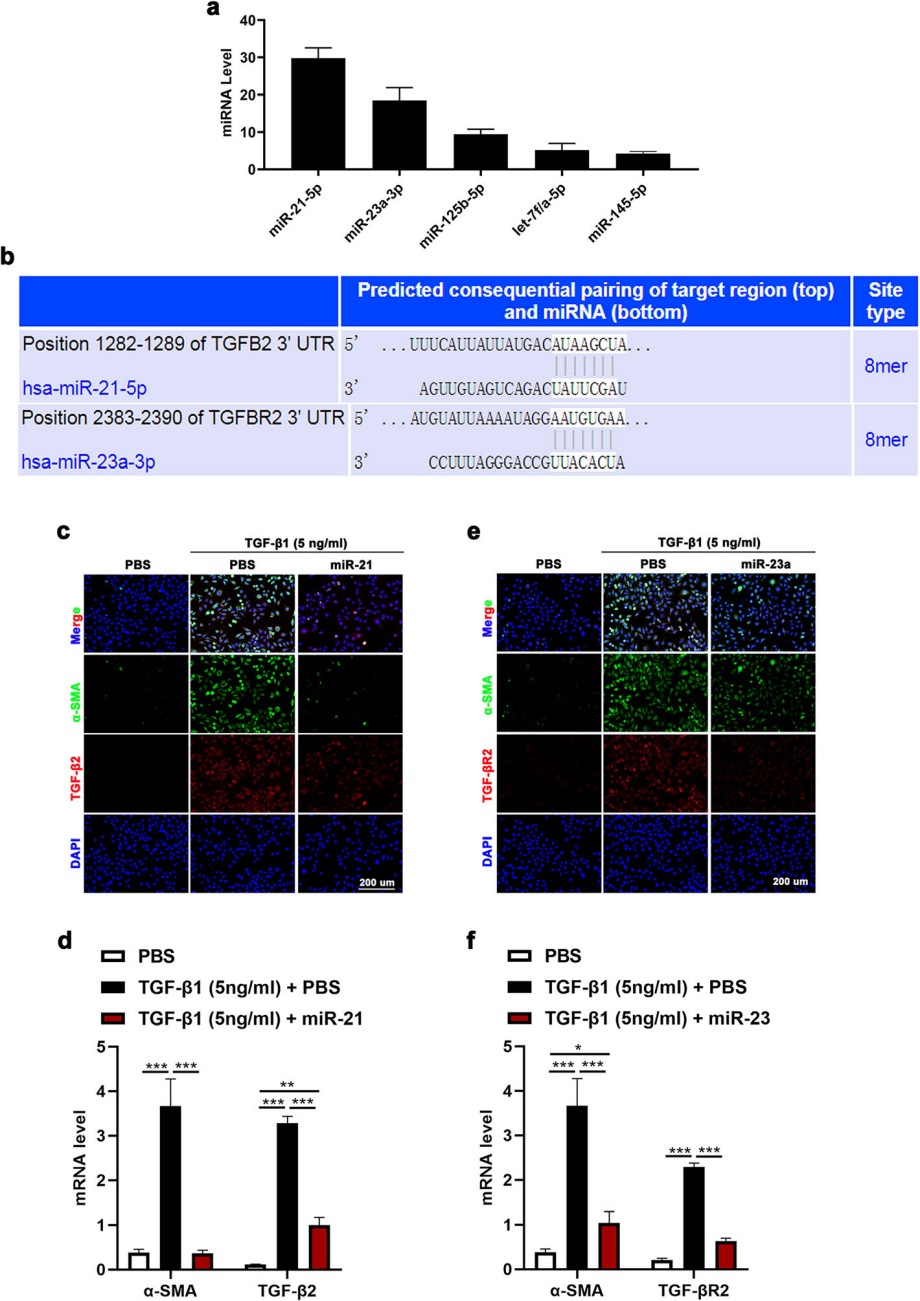
uMSC-EV-specific miRNAs target TGF-β2 and TGF-βR2 to inhibit myofibroblast differentiation. **a** Main miRNAs in uMSC-EVs using qRT-PCR. **b** A list of predicted binding sites of uMSC-EV-specific miRNAs and their targets. **c**–**e** Effect of uMSC-EV-specific miRNAs (mimics) on the expression levels of α-SMA, TGF-β2, and TGF-βR2 in TGF-β1-induced WML2 fibroblast cells (5 ng/mL) using IF and qRT-PCR. Scale bar = 200 μm. Mean ± SEM; *p < 0.05, **p < 0.01, ***p < 0.001; *n* = 3

The levels of miRNAs enriched in uMSC-EVs and their functions were investigated to determine the role of miRNAs in the uMSC-EVs in the inhibition of myofibroblast differentiation. We analyzed the sequencing results of miRNAs in uMSC-EVs reported by Fang et al. [10]. Results showed that miR-21-5p, miR-23a-3p, miR-125b-5p, let-7f/a, and miR-145-5p were highly expressed in uMSC-EVs (Fig. 7a). Then, the potential target genes of these miRNAs were predicted through Microrna (http://www.microrna.org) and TargetScan (http://www.targetscan.org/). Results showed that miR-21-5p and miR-23-3p were found to directly target TGF-β2 and TGF-βR2, respectively (Fig. 7b). Mimics were added in the WML2 fibroblast cell (+TGF-β1) culture system to determine whether or not they can directly affect the expression of TGF-β2 and TGF-βR2, and thus verify the role of these two miRNAs in uMSC-EV function. The results of IF staining and qRT-PCR analysis showed that miR-21 and miR-23 significantly inhibited the expression of TGF-β2 and TGF-βR2, respectively, and decreased the expression of α-SMA (Fig. 7c–f). Our results suggest that uMSC-EVs could effectively inhibit myofibroblast differentiation at least partially through miR-21-5p for TGF-β2 inhibition and miR-23-3p for TGF-βR2 inhibition.

## Discussion

MSC-based therapies reportedly reduce collagen deposition and inflammatory infiltration in PF treatment [35, 36]. Previous studies have reported that uMSC treatment could alleviate PF, but whether or not uMSCs participate in tissue repair directly by themselves or indirectly by their paracrine factors has not yet been clearly explained [17, 37]. In this work, we demonstrated that uMSC-EVs showed similar effects on BLM-induced PF mice as the uMSCs, which increased the survival rate, improved the weight loss and the destruction of normal lung tissue architecture and dense deposition of collagen, and stimulated the proliferation of lung epithelial cells, revealing for the first time the therapeutic effects of uMSCs in PF, which can be, at least in part, via uMSC-EVs. To a certain extent, the present report provides a new insight for PF treatment in the clinic using uMSCs.

The umbilical cord is an acceptable, economical, and efficient source of MSCs. Compared with bone marrow-derived MSCs and adipose-derived MSCs, uMSCs have more advantages due to their stronger proliferation and differentiation abilities [37, 38]. The function of MSCs in cell therapy on tissue injury, including immunomodulation, anti-inflammatory activity, anti-apoptotic activity, and angiogenesis regulation, might be realized through paracrine pathway. As reviewed elsewhere [10, 26, 28], most of these effects are mediated by their secretory EVs. Consistently, we observed that after intravenous injection of PKH67-labeled uMSCs into PF mice, the majority of uMSCs could be detected within 1–7 days. However, only very few uMSCs were observed on day 14 (Fig. S2), indicating that their paracrine activities exert the main therapeutic effects on uMSCs, rather than that uMSCs per se participating in the formation of new tissues.

EVs contain a cell-specific cargo of nucleic acids, proteins, and lipids to modulate the activity of recipient cells and play essential roles in tissue injury [31, 32]. Because of enveloped with a lipid bilayer, the EV-carried “cargoes” are protected from degradation, suggesting that it has advantages for storage and transport. Interestingly, in the present study, uMSC-EVs could be detected on days 1–7 after injection into the PF mice (Fig. S3), suggesting that uMSC-EVs can function in the body stably within 1 week after treatment. Furthermore, application of the EVs as a cell-free therapy provides crucial advantages over stem cell therapies [39], (1) can avoid many risks associated with the transplantation of living cell, including tumorigenicity, immune compatibility, and the transmission of infections; (2) can be evaluated for safety, potency, and dosage in a more accurate manner; (3) can be stored without toxic cryopreservative agents for an extended period with product potency; (4) can be mass-produced through tailor-made cell lines; and (5) can obtain the desired specific EVs through modifying the parent cells. However, there are still some problems in EV applications; their purity and yield restrict their ready clinical application. In the present study, we used differential ultracentrifugation to prepare uMSC-EVs, the most widely used laboratory methodology [40]. However, this method might lead to aggregation and co-precipitation with soluble proteins presented in the biofluid or even cause vesicle rupture or fusion with contaminants and other proteins [41]. Moreover, this method is undoubtedly challenging to translate into the clinic given their time-consuming costs, low yield, requiring a specialized ultracentrifuge, and lack of automatization [42]. Alternative procedures have been explored and tried to allow purer EV preparations with easy implementation. The most practical one is the size exclusion chromatography (SEC), which removes most of the overabundant soluble proteins, reduces time-consuming, and maintains the major EVs’ characteristic in EV preparation [43]. If uMSC-EVs are used to treat PF in the clinic in the future, further improved SEC may be the best choice for preparing EVs.

Myofibroblasts are the primary collagen-producing cells in PF [1–3]. FMT lung fibroblasts in situ are the main contributor to myofibroblasts [44, 45]. Consistent with these findings, we observed that the lung tissues of PF mice exhibited increased myofibroblast markers α-SMA and Fn. Notably, uMSC-EV treatment significantly reduced the expression levels of α-SMA and Fn. Similarly, the FMT model of WML2 fibroblast cells induced by TGF-β1 in vitro showed that uMSC-EVs reduced the high expression level of α-SMA. TGF-β1, which activates the TGF-β signaling pathway, is reportedly a pivotal factor in producing α-SMA and stimulating FMT [5, 6]. However, direct TGF-β-based antifibrotic therapy has an adverse immune response [46, 47]. Therefore, intervening with the critical upstream effectors of the TGF-β pathway provides a therapeutic target for PF. uMSC-EVs decreased the expression levels of TGF-β signaling pathway-related genes, including TGF-β2 and TGF-βR2 in vivo (Fig. 5) and in vitro (Fig. 6). These findings suggest that uMSC-EVs in PF treatment may specifically target TGF-β2 and TGF-βR2 to inhibit myofibroblast differentiation. Besides, we also detected the expression of TGF-β1, TGF-β3, and TGF-βR1 using IF staining of lung tissues in mice (Fig. S4) and found that there was no significant difference in TGF-β3 expression among the groups; however, TGF-β1 and TGF-βR1 were highly expressed in the lung tissue of the PF mice. Both uMSC and uMSC-EV treatments reduced the expression level of TGF-β1 but had no significant effect on TGF-βR1. In the present study, we mainly focused on TGF-β2 and TGF-βR2; thus, the expression status of TGF-β1 needs further research in the future. Furthermore, we found that uMSCs serve a critical role in PF alleviation and myofibroblast differentiation inhibition through their derived miRNAs. The FMT suppression function of uMSC-EVs-miRNAs might be associated with their targeted roles for the expression of TGF-β2 and TGF-βR2 inhibition. The results of the present study showed that miR-21-5p, miR-23a-3p, miR-125b-5p, let-7f/a, and miR-145-5p were enriched in uMSC-EVs. The target genes of these miRNAs were predicted using TargetScan (http://www.targetscan.org/) and Microrna (http://www.microrna.org), and results revealed that miR-21-5p and miR-23-3p directly target TGF-β2 and TGF-βR2, respectively. Then, mimics were used for verification, and results showed that miR-21 inhibited TGF-β2 and miR-23 inhibited TGF-βR2 expression significantly (Fig. 7). MiR-21-5p and miR-23-3p have been previously reported to suppress fibrotic diseases, including wound scar formation [10], liver fibrosis [48], and kidney fibrosis [49], which is consistent with our present findings. The repair mechanism of miRNAs in different organs and tissues possibly is similar. Therefore, the anti-FMT effects of miR-21-5p and miR-23-3p in uMSC-EVs may help us to understand the functions of miRNAs. These uMSC-EV-specific miRNAs could be critical inhibitors of the TGF-β signaling pathway, suppressing FMT in pulmonary fibrogenesis.

Overall, the present study focused on the antifibrotic effects of uMSC-EVs. However, the immunomodulatory effect of MSCs also takes part in tissue repair [28]. Recent studies reported that intravenous administration of MSCs elicits an immunomodulatory response through polarization of macrophages [50], induction of regulatory T cells [51], and producing anti-inflammatory cytokines [52]. Therefore, whether uMSC-EVs could recapitulate the immunomodulatory effects of their parent cells is deserved to investigate in the future study. Also, the optimal dosage, timing, and delivery route of uMSC-EVs are needed to be investigated.

## Conclusion

**Fig. 8:**
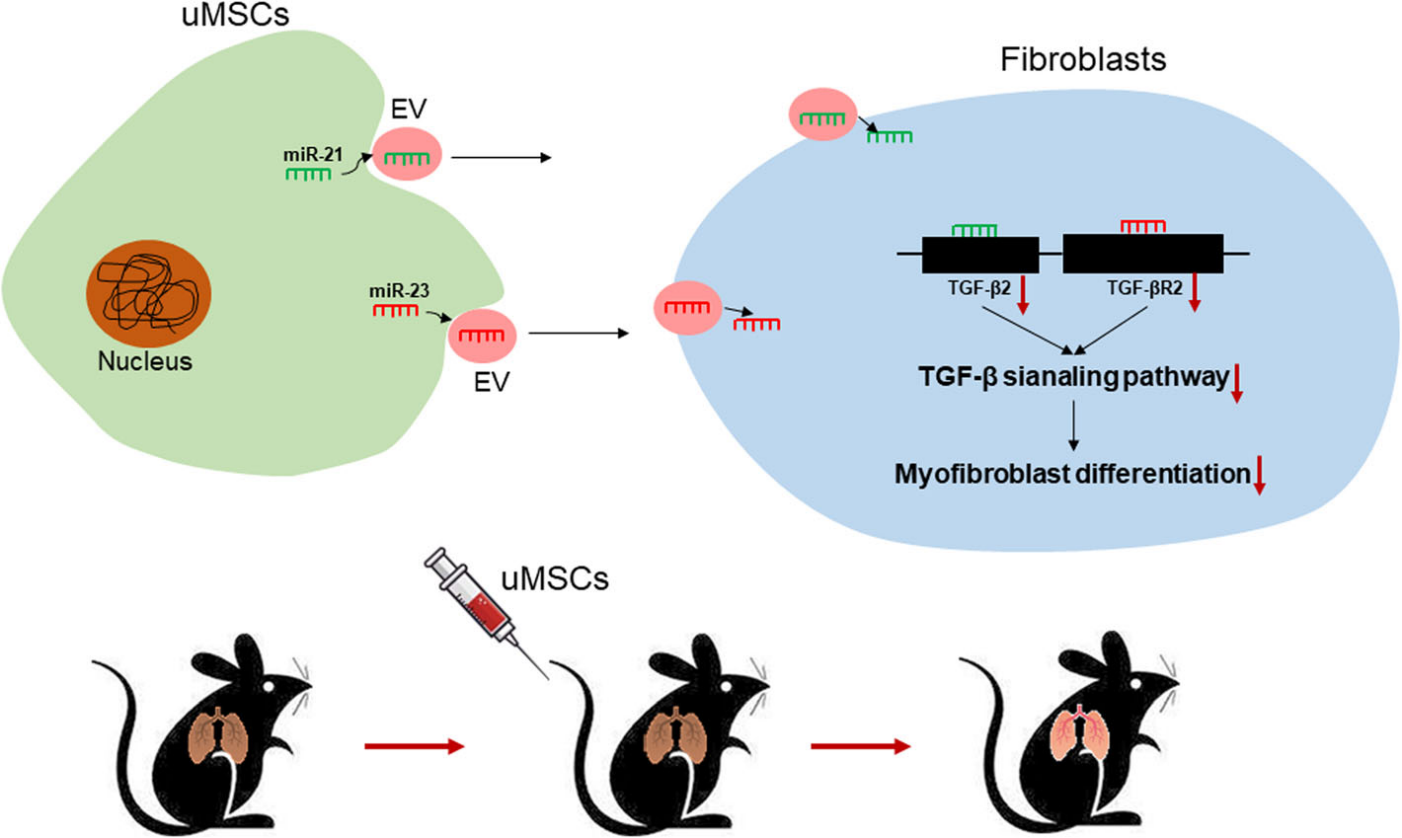
uMSC-EVs alleviated pulmonary fibrosis by inhibiting myofibroblast differentiation. MiR-21 and miR-23 carried by uMSC-EVs might inhibit the differentiation of fibroblasts into myofibroblasts by downregulating the expression of TGF-β 2 and TGF-β R2 in the TGF-β signaling pathway

This study in the first time revealed the therapeutic effects of uMSCs on PF likely through their secreted EVs. uMSC-EV-derived miR-21-5p and miR-23-3p effectively suppressed the TGF-β signaling pathway via inhibiting TGF-β2 and TGF-βR2 expression (Fig. 8). As an alternative strategy for stem cell therapy, uMSC-EVs could become a new advantageous clinical method for PF treatment.

## Supplementary Information

**Additional file 1: Figure S1.** Effect of different concentrations of TGF-β1 on α-SMA expression. WML2 fibroblast cells were cultured for 48 h with different concentrations of TGF-β1 (0, 0.25, 5, and 10 ng/mL) to induce myofibroblast differentiation, and then the expression levels of α-SMA were detected in different treatments using IF **(a)** and qRT-PCR **(b)**. Scale bar = 200 μm. Mean ± SEM; ^***^p < 0.001; *n* = 3. TGF-β1, transforming growth factor-β1; α-SMA, α-smooth muscle actin; Fn, fibronectin. **Figure S2.** uMSC lineage tracing in the lung tissue of BLM-induced mouse. uMSCs were labeled with PKH67 and then injected via mouse tail veins of BLM-induced mouse. uMSC tracing in the lung tissue was performed on days 1, 4, 7, and 14 after cell injection. **Figure S3.** uMSC-EVs lineage tracing in the lung tissue of BLM-induced mouse. uMSC-EVs were labeled with PKH67 and then injected via mouse tail veins of BLM-induced mouse. uMSC-EVs-tracing in the lung tissue were performed on days 1, 4, 7, and 14 after cell injection. **Figure S4.** Effects of uMSC-EVs on the expression levels of TGF-β1, TGF-β3 and TGF-βR1 in lung tissues of PF mice. **a** Expression levels of TGF-β1. **b** Expression levels of TGF-β3. **c** Expression levels of TGF-βR1. Scale bar = 200 μm. **Table S1.** Antibodies used in the surface antigen expression detection of uMSCs. **Table S2.** Primers.

## Data Availability

The datasets used and/or analyzed during the present study are available from the corresponding author on reasonable request.

## Abbreviations

α-SMA: α-Smooth muscle actin
BLM: Bleomycin
FMT: Fibroblast-to-myofibroblast transition
Fn: Fibronectin
HYP: Hydroxyproline
TGF-β1: Transforming growth factor-β1
TGF-β2: Transforming growth factor-β2
TGF-βR2: Transforming growth factor-β receptor type II
uMSCs: Umbilical cord-derived mesenchymal stromal cells
uMSC-EVs: Umbilical cord-derived mesenchymal stromal cell-derived extracellular vesicles
