# Supplementary material for: Extracellular vesicles derived from umbilical cord mesenchymal stromal cells alleviate pulmonary fibrosis by means of transforming growth factor-β signaling inhibition
Source: Stem Cell Res Ther. 2021 Apr 12;12:230. doi: 10.1186/s13287-021-02296-8 (PMC8041243; doi:10.1186/s13287-021-02296-8)
Supplement: Supplementary file 1 — Additional file 1: Figure S1. Effect of different concentrations of TGF-β1 on α-SMA expression. WML2 fibroblast cells were cultured for 48 h with different concentrations of TGF-β1 (0, 0.25, 5, and 10 ng/mL) to induce myofibroblast differentiation, and then the expression levels of α-SMA were detected in different treatments using IF (a) and qRT-PCR (b). Scale bar = 200 μm. Mean ± SEM; ***p < 0.001; n = 3. TGF-β1, transforming growth factor-β1; α-SMA, α-smooth muscle actin; Fn, fibronectin. Figure S2. uMSC lineage tracing in the lung tissue of BLM-induced mouse. uMSCs were labeled with PKH67 and then injected via mouse tail veins of BLM-induced mouse. uMSC tracing in the lung tissue was performed on days 1, 4, 7, and 14 after cell injection. Figure S3. uMSC-EVs lineage tracing in the lung tissue of BLM-induced mouse. uMSC-EVs were labeled with PKH67 and then injected via mouse tail veins of BLM-induced mouse. uMSC-EVs-tracing in the lung tissue were performed on days 1, 4, 7, and 14 after cell injection. Figure S4. Effects of uMSC-EVs on the expression levels of TGF-β1, TGF-β3 and TGF-βR1 in lung tissues of PF mice. a Expression levels of TGF-β1. b Expression levels of TGF-β3. c Expression levels of TGF-βR1. Scale bar = 200 μm. Table S1. Antibodies used in the surface antigen expression detection of uMSCs. Table S2. Primers. [file 13287_2021_2296_MOESM1_ESM.docx]

**Supplementary materials**


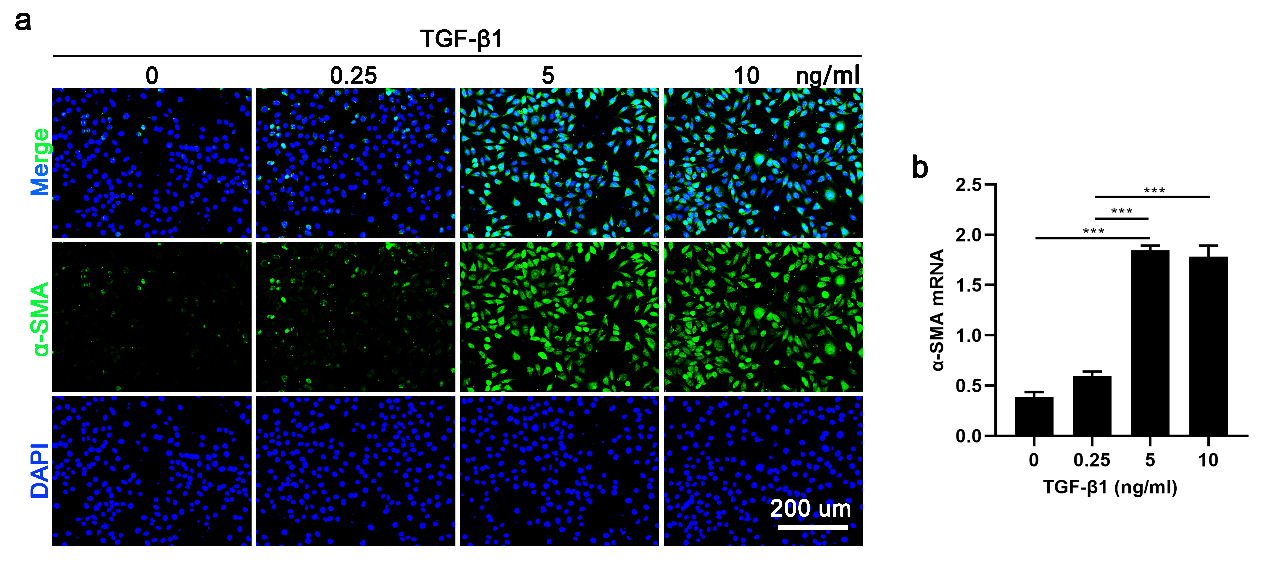


**Figure S1** Effect of different concentrations of TGF-β1 on α-SMA expression. WML2 fibroblast cells were cultured for 48 h with different concentrations of TGF-β1 (0, 0.25, 5, and 10 ng/mL) to induce myofibroblast differentiation, and then the expression levels of α-SMA were detected in different treatments using IF **(a)** and qRT-PCR **(b)**. Scale bar = 200 μm. Mean ± SEM; ^***^*p*  <  0.001; n  =  3. TGF-β1, transforming growth factor-β1; α-SMA, α-smooth muscle actin; Fn, fibronectin.


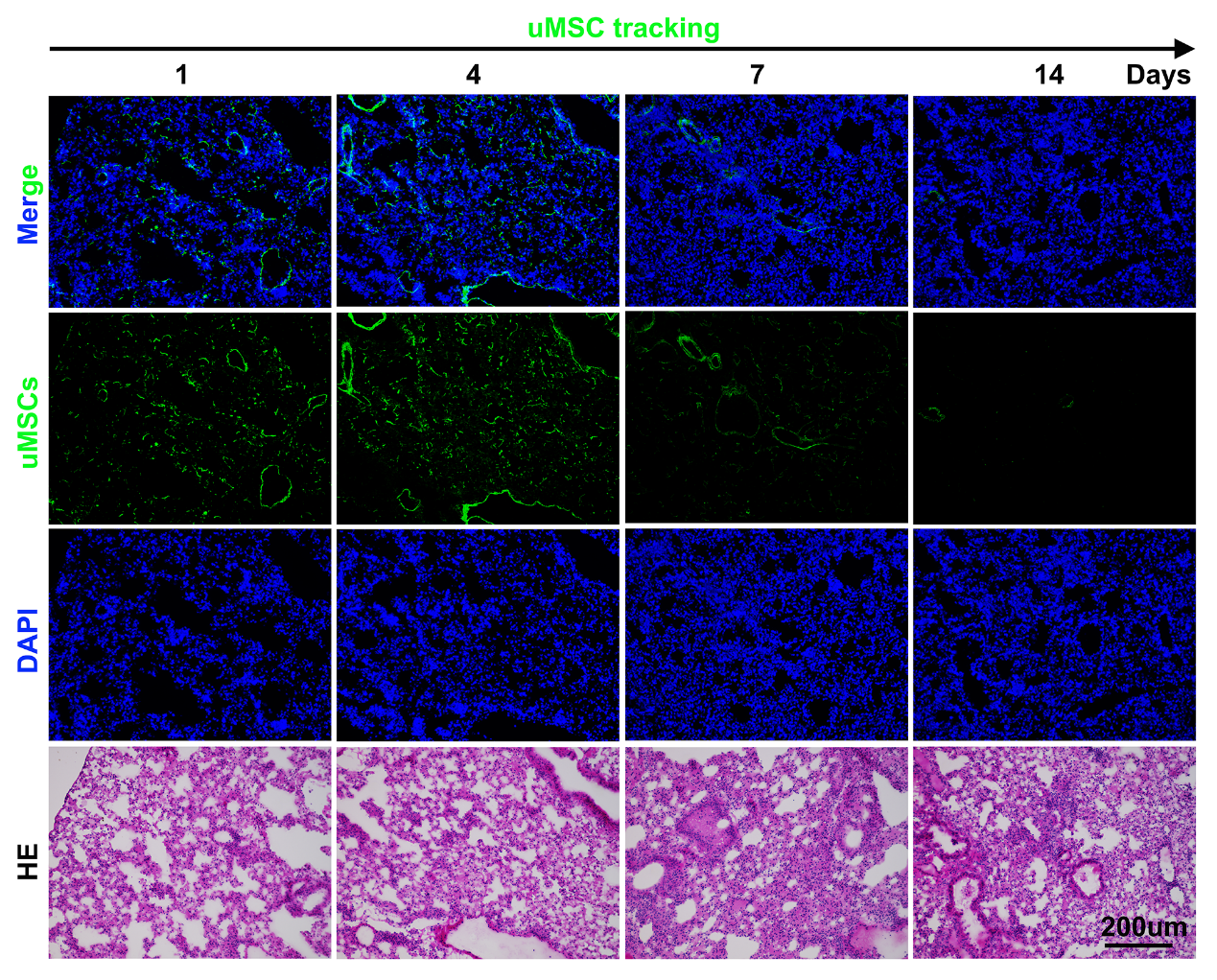


**Figure S2** uMSC lineage tracing in the lung tissue of BLM-induced mouse. uMSCs were labeled with PKH67 and then injected via mouse tail veins of BLM-induced mouse. uMSC tracing in the lung tissue was performed on days 1, 4, 7, and 14 after cell injection.


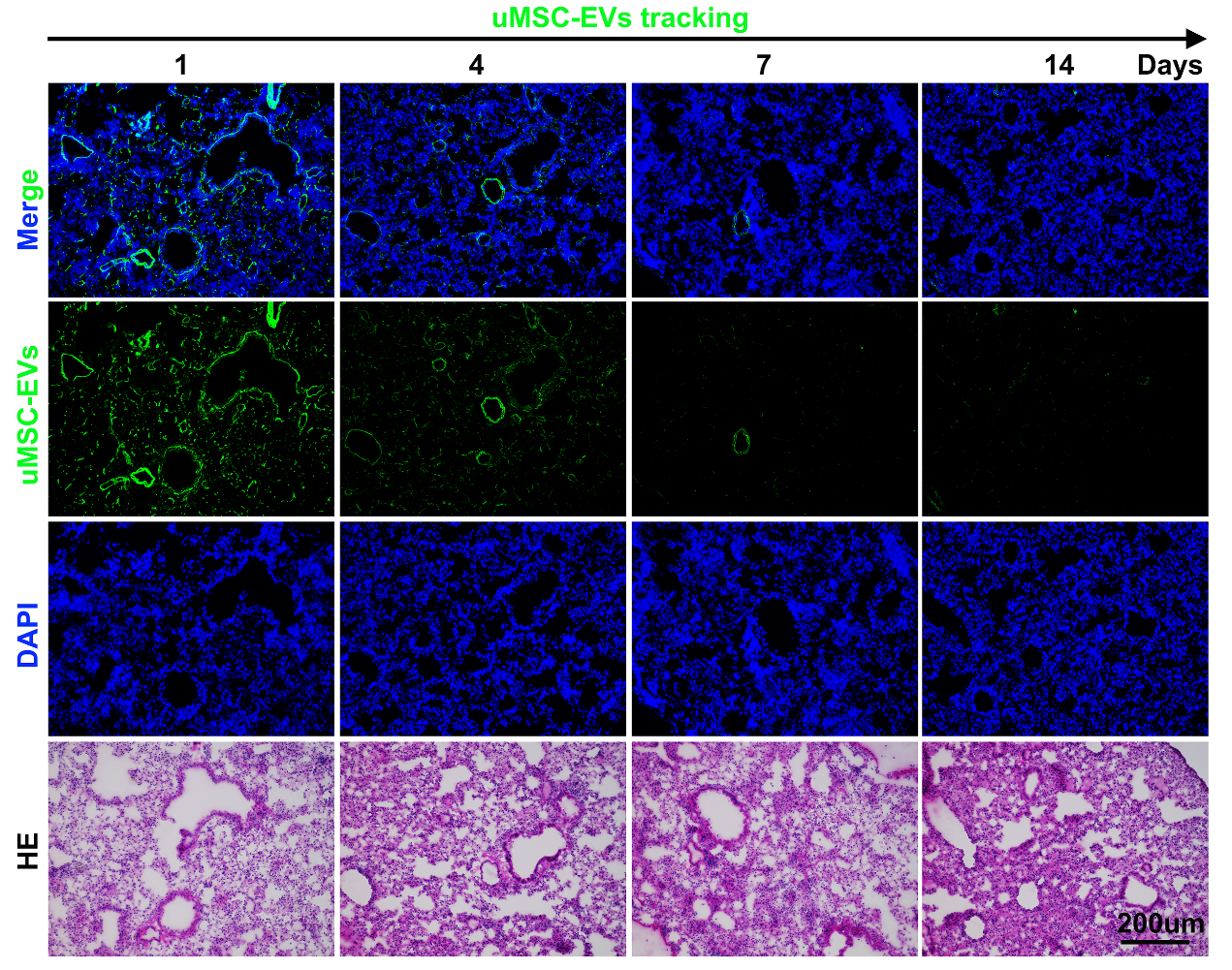


**Figure S3** uMSC-EVs lineage tracing in the lung tissue of BLM-induced mouse. uMSC-EVs were labeled with PKH67 and then injected via mouse tail veins of BLM-induced mouse. uMSC-EVs-tracing in the lung tissue were performed on days 1, 4, 7, and 14 after cell injection.


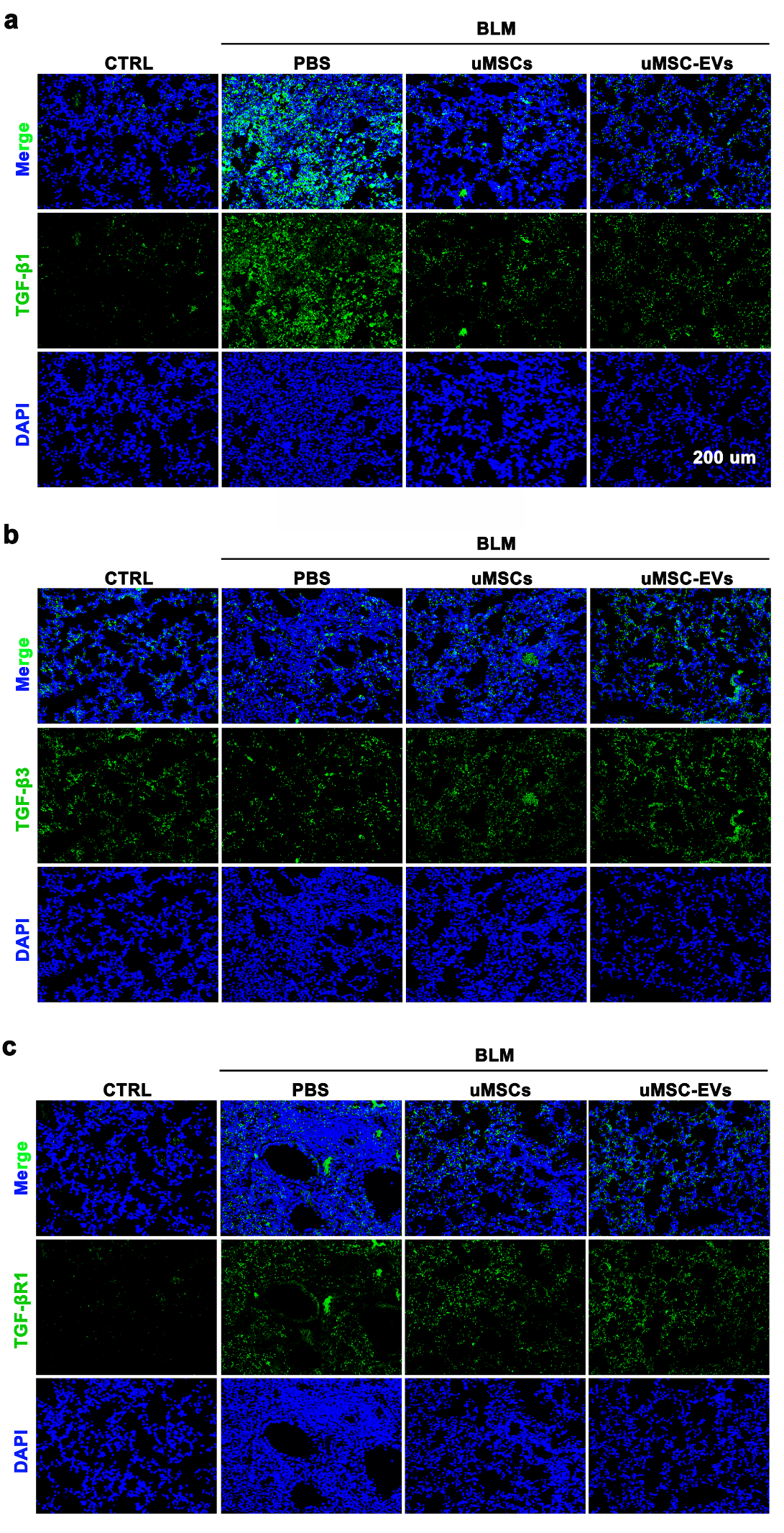


**Figure S4** Effects of uMSC-EVs on the expression levels of TGF-β1, TGF-β3 and TGF-βR1 in lung tissues of PF mice. **a** Expression levels of TGF-β1. **b** Expression levels of TGF-β3. **c** Expression levels of TGF-βR1. Scale bar = 200 μm.

**Table S1** Antibodies used in the surface antigen expression detection of uMSCs

| **Antibody** | **Company** | **Catalog number** | **Dilution** |
| --- | --- | --- | --- |
| anti-CD34 | Bioss, China | bs-0646R | 1:500 (IF); 1:100 (FCM) |
| anti-CD45 | Bioss, China | bs-4819R | 1:500 (IF); 1:100 (FCM) |
| anti-CD73 | Bioss, China | bs-4834R | 1:500 (IF); 1:100 (FCM) |
| anti-CD90 | Bioss, China | bs-20640R | 1:500 (IF); 1:100 (FCM) |
| anti-CD105 | Bioss, China | bs-0579R | 1:500 (IF); 1:100 (FCM) |

**Table S2** Primers

| **qRT-PCR primers** | | |
| --- | --- | --- |
| α-SMA | F | AGCCATGTACGTAGCCATCC |
|  | R | CTCTCAGCTGTGGTGGTGAA |
| TGF-β2 | F | TTGTGCTCCAGACAGTCCCA |
|  | R | ATCCGTTGTTCAGGCACTCT |
| TGF-βR2 | F | CTGCCCATCCACTGAGACATA |
|  | R | AGCTTGGGGTCATGGCAAAC |
| Fibronectin | F | GGATCCCCTCCCAGAGAAGT |
|  | R | GGGTGTGGAAGGGTAACCAG |
| GAPDH | F | TGCCCCCATGTTTGTGATG |
|  | R | TGTGGTCATGAGCCCTTCC |
| hsa-miR-21-5p-RT | Reverse transcription primer | GTCGTATCCAGTGCGAACTGTGGCGATCGGTACGGGCTACACTCGGCAATTGCACTGGATACGACtcaac |
| hsa-miR-21-5p-RealR | R | GCGTAGCTTATCAGACTGATG |
| hsa-miR-23a-3p-RT | Reverse transcription primer | GTCGTATCCAGTGCGAACTGTGGCGATCGGTACGGGCTACACTCGGCAATTGCACTGGATACGACggaaa |
| hsa-miR-23a-3p-RealR | R | CGATCACATTGCCAGGGAT |
| hsa-miR-125b-5p-RT | Reverse transcription primer | GTCGTATCCAGTGCGAACTGTGGCGATCGGTACGGGCTACACTCGGCAATTGCACTGGATACGACtcaca |
| hsa-miR-125b-5p-RealR | R | CCTCCCTGAGACCCTAACTT |
| hsa-let-7a/f-5p-RT | Reverse transcription primer | GTCGTATCCAGTGCGAACTGTGGCGATCGGTACGGGCTACACTCGGCAATTGCACTGGATACGACaacta |
| hsa-let-7a/f-5p-RealR | R | GCGCTGAGGTAGTAGATTGTAT |
| hsa-miR-145-5p-RT | Reverse transcription primer | GTCGTATCCAGTGCGAACTGTGGCGATCGGTACGGGCTACACTCGGCAATTGCACTGGATACGACaggga |
| hsa-miR-145-5p-RealR | R | CCGTCCAGTTTTCCCAGGAA |
| Uni-Primer | F | AGTGCGAACTGTGGCGAT |
